# Harmonization of Multicenter Cortical Thickness Data by Linear Mixed Effect Model

**DOI:** 10.3389/fnagi.2022.869387

**Published:** 2022-06-17

**Authors:** SeungWook Kim, Sung-Woo Kim, Young Noh, Phil Hyu Lee, Duk L. Na, Sang Won Seo, Joon-Kyung Seong

**Affiliations:** ^1^Department of Bio-Convergence Engineering, Korea University, Seoul, South Korea; ^2^Department of Neurology, Gil Medical Center, Gachon University College of Medicine, Incheon, South Korea; ^3^Department of Neurology, Yonsei University College of Medicine, Seoul, South Korea; ^4^Department of Neurology, Samsung Medical Center, Sungkyunkwan University School of Medicine, Seoul, South Korea; ^5^Neuroscience Center, Samsung Medical Center, Seoul, South Korea; ^6^Samsung Alzheimer Research Center, Center for Clinical Epidemiology, Samsung Medical Center, Seoul, South Korea; ^7^Department of Health Sciences and Technology, Clinical Research Design and Evaluation, SAIHST, Sungkyunkwan University, Seoul, South Korea; ^8^School of Biomedical Engineering, Korea University, Seoul, South Korea; ^9^Department of Artificial Intelligence, Korea University, Seoul, South Korea; ^10^Interdisciplinary Program in Precision Public Health, Korea University, Seoul, South Korea

**Keywords:** magnetic resonance imaging, cortical thickness, multicenter data harmonization, linear mixed effect model, Alzheimer’s disease, Parkinson’s disease

## Abstract

**Objective:**

Analyzing neuroimages being useful method in the field of neuroscience and neurology and solving the incompatibilities across protocols and vendors have become a major problem. We referred to this incompatibility as “center effects,” and in this study, we attempted to correct such center effects of cortical feature obtained from multicenter magnetic resonance images (MRIs).

**Methods:**

For MRI of a total of 4,321 multicenter subjects, the harmonized w-score was calculated by correcting biological covariates such as age, sex, years of education, and intercranial volume (ICV) as fixed effects and center information as a random effect. Afterward, we performed classification tasks using principal component analysis (PCA) and linear discriminant analysis (LDA) to check whether the center effect was successfully corrected from the harmonized w-score.

**Results:**

First, an experiment was conducted to predict the dataset origin of a random subject sampled from two different datasets, and it was confirmed that the prediction accuracy of linear mixed effect (LME) model-based w-score was significantly closer to the baseline than that of raw cortical thickness. As a second experiment, we classified the data of the normal and patient groups of each dataset, and LME model-based w-score, which is biological-feature-corrected values, showed higher classification accuracy than the raw cortical thickness data. Afterward, to verify the compatibility of the dataset used for LME model training and the dataset that is not, intraobject comparison and w-score RMSE calculation process were performed.

**Conclusion:**

Through comparison between the LME model-based w-score and existing methods and several classification tasks, we showed that the LME model-based w-score sufficiently corrects the center effects while preserving the disease effects from the dataset. We also showed that the preserved disease effects have a match with well-known disease atrophy patterns such as Alzheimer’s disease or Parkinson’s disease. Finally, through intrasubject comparison, we found that the difference between centers decreases in the LME model-based w-score compared with the raw cortical thickness and thus showed that our model well-harmonizes the data that are not used for the model training.

## Introduction

Analyzing neuroimages has been a useful method in the field of neuroscience and neurology. Previous studies have shown that magnetic resonance imaging (MRI) can be used to find meaningful biomarkers for various clinical diseases such as Alzheimer’s disease (AD) (Frisoni et al., 2010; Davatzikos et al., 2011; Salvatore et al., 2015), Parkinson’s disease (PD) (Schwarz et al., 2011; Salvatore et al., 2014), brain tumors (Arevalo-Perez et al., 2015), and so on. Specifically, cortical thickness has contributed to reveal clinical features and content of such neurodegenerative diseases (Querbes et al., 2009; Gao et al., 2018; Wannan et al., 2019). For example, predicting mild cognitive impairment (MCI) or AD conversion through normalized thickness in longitudinal data is a typical usage of cortical thickness (Querbes et al., 2009). In addition, the relationship between cortical reduction in specific brain regions and PD disease severity at different Hoehn-Yahr (H-Y) stages is found using cortical thickness (Gao et al., 2018). The brain cortical thickness network analysis informs the irregular topographic distribution of cortical thickness reduction in schizophrenia (Wannan et al., 2019).

There are many cases of applying machine learning to the neuroimage field (Moradi et al., 2015; Ball et al., 2016; Steele et al., 2017). Among them, remarkable results came from studies using cortical thickness as the learning features (Eskildsen et al., 2014; Lavagnino et al., 2018). It is difficult to deny that one of the most important points in a machine learning study is the number of training data, but it can be seen that a large number of neuroimage studies only used datasets acquired from a single center. This is because there is no unified protocol for acquiring T1 images across multiple centers and vendors; there is variety of scanner types in the field, and such heterogeneity of protocols and vendors creates incompatibilities between the acquired images (Kruggel et al., 2010). In this article, we call these incompatibilities across protocols and vendors “center effect.”

Several methods have been proposed to solve the center effect problem (Chung et al., 2017; Fortin et al., 2018; Zhao et al., 2019; Sun et al., 2021). Among then, two of the most representatives are combat harmonization (Johnson et al., 2007; Fortin et al., 2018) and w-score method (Chung et al., 2017). Both methods are based on multiple linear regression (MLR), and they are shown to be able to compute good-quality standard scores on data in centers with sufficient number of cognitive normal (CN) training data. However, this method imposes the following limitations on the problem of calculating the harmonization score when acquiring data from a new center: (1) A lack of CN data in a new center cannot train a harmonization model, or even if trained, the harmonization score calculated by that model cannot be trusted. (2) Even if the number of CN data for the new center is sufficient, the harmonization model, including the center, should be retrained from the beginning, or a new harmonization model for the center should be trained separately. Therefore, as new data are added, it is impossible for the existing harmonization model to grow through the reinforcement process.

In this study, we introduced a harmonization technique based on the linear mixed effect (LME) model to overcome these limitations. The LME model has mainly been used for the correction or analysis of time points in longitudinal studies (Bernal-Rusiel et al., 2013a,b). In our problem setting, we used the LME model to explore multicenter cortical thickness measurements by setting the center information as the random effects of the model instead of time points (Ten Kate et al., 2018; Laansma et al., 2021). We first demonstrated the efficacy of the proposed LME method compared with two other harmonization methods using a total of 10 discovery datasets divided according to various scanner types and protocols. We showed that the score calculated by the LME method effectively compensates the center effect across multiple datasets, preserves the disease effect, and has the scalability of the model compared with the other two harmonization methods.

The contributions of our harmonization model are as follows:

1. Our model can express the cortical thickness extracted from T1-MRI as a center effect-free normalized w-score, which represents the degree of regional cortical atrophy.
2. Our model can calculate the w-score for a subject from a center that cannot build the cortical atrophy model on its own due to the insufficient number of CN individuals of the corresponding center.
3. Even when data of centers with enough CN individuals are added, the LME model can be updated without the whole training process similar to the framework of online learning.

## Materials and Methods

### Participants

There are total 4,321 T1-weighted MRIs in the discovery set, including 3,641 CN subjects, 823 AD patients, and 81 PD patients. Among them, 537 CN and 343 AD subjects are collected from the Alzheimer’s Disease Neuroimaging Initiative (ADNI); 75 CN and 56 AD subjects are collected from Open Access Series of Imaging Studies (OASIS); 2,907 CN and 351 AD subjects are collected from the Samsung Medical Center (SMC); 51 CN and 64 AD subjects are collected from the Gacheon Medical Center (GMC); and 71 CN and 81 PD subjects are collected from the Shinchon Severence Hospital. As an external validation set, images of 10 subjects were acquired at SMC and Chaum Hospital at similar time.

According to the field strength and manufacture of MRI scanners, we divided the ADNI dataset into a total of 6 datasets. By combining the remaining centers, total 10 independent datasets were used as a discovery set in this study. The criteria for dividing all the datasets (10 discovery set + 1 external validation set) are described in Table 1. The descriptive statistics of each dataset are provided at Table 2.

**TABLE 1:** Scan parameters for T1-weighted magnetic resonance imaging (MRI) of each dataset.

Dataset	No. of subjects	Center/ Cohort	Manufacturer	Field Strength (Tesla)	TR^a^ (ms)	TE^b^ (ms)	Flip angle (°)
D1	203	ADNI	GE^c^	1.5	3000	100	8
D2	140	ADNI	GE	3	3000	97.2	8
D3	41	ADNI	Philips	1.5	shortest	4	8
D4	102	ADNI	Philips	3	shortest	shortest	8
D5	167	ADNI	Siemens	1.5	2400	3.5	8
D6	227	ADNI	Siemens	3	2300	2.91	9
D7	131	OASIS	Siemens	1.5	9.7	4	10
D8	3258	SMC	Philips	3	9.9	4.6	8
D9	115	GMC	Siemens	3	1900	2.93	8
D10	152	Severance	Philips	3	9.8	4.6	8
D11	10	Chaum	GE	3	9.12	3.568	12

*^a^Repetition time.*

*^b^Echo time.*

*^c^General Electric Healthcare.*

**TABLE 2:** Description of the subjects of each dataset set.

Dataset	Group	No. of subjects	No. of males (%)	Age^a^	Years of Education^b^	ICV^c^ (x10^5^)
D1	CN	107	55 (51)	76.15 ± 4.56	15.84 ± 3.01	15.32 ± 1.54
	AD	96	49 (51)	74.87 ± 7.84	14.74 ± 3.21	15.37 ± 1.85
D2	CN	92	36 (39)	72.83 ± 5.67	16.48 ± 2.67	14.32 ± 1.18
	AD	48	29 (60)	74.50 ± 8.15	15.48 ± 2.91	14.60 ± 1.65
D3	CN	25	18 (72)	74.76 ± 3.57	17.24 ± 2.20	15.62 ± 1.18
	AD	16	7 (44)	74.20 ± 9.20	14.63 ± 3.30	15.21 ± 1.69
D4	CN	68	29 (43)	72.99 ± 6.04	16.63 ± 2.44	14.96 ± 2.18
	AD	34	16 (47)	72.54 ± 7.12	15.76 ± 2.88	15.29 ± 2.34
D5	CN	93	42 (45)	76.03 ± 5.79	15.91 ± 2.76	15.31 ± 1.73
	AD	74	36 (49)	76.97 ± 7.20	14.49 ± 3.39	15.44 ± 1.78
D6	CN	152	76 (50)	73.03 ± 6.40	16.74 ± 2.50	15.01 ± 1.56
	AD	75	45 (60)	75.73 ± 8.09	15.96 ± 2.58	15.43 ± 1.65
D7	CN	75	21 (28)	74.65 ± 7.92	15.28 ± 2.73	14.54 ± 1.59
	AD	56	29 (52)	75.43 ± 6.55	13.71 ± 2.83	14.50 ± 1.70
D8	CN	2907	1455 (50)	64.12 ± 7.20	12.76 ± 4.33	12.48 ± 2.09
	AD	351	111 (32)	71.21 ± 9.23	9.17 ± 5.59	13.87 ± 2.00
D9	CN	51	27 (53)	64.24 ± 11.30	11.80 ± 4.84	13.99 ± 1.86
	AD	64	21 (33)	66.33 ± 10.09	8.96 ± 4.70	13.82 ± 1.93
D10	CN	71	28 (39)	65.89 ± 7.57	12.79 ± 4.33	12.24 ± 2.20
	PD	120	59 (49)	64.70 ± 7.25	10.70 ± 5.03	13.08 ± 2.21
D11	–	10	5 (50)	72.2 ± 8.80	–	13.69 ± 1.86

*^a^Mean ± SD (range), years.*

*^b^Mean ± SD (range), years.*

*^c^Intracranial volume, Mean ± SD (range), mm^3^.*

### Image Acquisition and Preprocessing

Magnetic resonance imaging scans were performed under various scanner conditions, including different Tesla (1.5 and 3.0 T), manufacturers (GE Healthcare, Philips Medical Systems or Siemens Medical Solutions), TR, TE, etc. The summaries of scan parameters for each center are also described at Table 1 (Jack et al., 2008; Marcus et al., 2010; Cho et al., 2016; Chung et al., 2017, 2019; Jeong et al., 2020).

All images underwent preprocessing steps performed with the standard FreeSurfer T1 MRI preprocessing pipeline^1^. For the intensity-scale standardization, the raw image was conformed to the common voxel size to control image resolution. Then, nonparametric and non-uniform intensity normalization was performed (Sled et al., 1998). A series of intensity normalization steps were performed to improve the intensity-based segmentation. After the intensity-scale standardization, white matter and pial surfaces were segmented, and cortical thickness was measured at every vertex. Finally, the mean cortical thickness values were extracted for 68 regions of interest (ROIs) defined by the Desikan-Kiliany atlas.

During preprocessing steps, intracranial volume (ICV) was calculated for each subject. ICV is defined as the total brain volume, including white matter, gray matter, cerebrospinal fluid, and meninges. We used ICV as a biological covariate to measure the individual variability, just like age, sex, and years of education.

### Harmonization Procedures

For the harmonization procedures, we compared three different methods, namely, (1) protocol-specific w-score (Chung et al., 2017) referred as *Self-W*; (2) *ComBat* (Johnson et al., 2007); and (3) LME model-based w-score referred as *LME-W*. We also compared the absence of the harmonization procedure, which we refer to as *raw*. Each harmonization techniques are described in the following sections.

#### Protocol-Specific W-Score

W-score is a standardized score of disease values compared with the distribution of normal values, using MLR (Chung et al., 2017). The protocol-specific w-score is modeled under the assumption that the observed cortical thickness is predictable by biological features. However, Chung et al. (2017) assumed that the values corresponding to each region in a subject’s brain are nonlinearly distributed. In addition, the difference according to the imaging protocol of each dataset is also assumed to be nonlinear, and these nonlinear effects work equally for the same region of patients obtained under the same protocol. In other words, the protocol specific w-score models the following equation with the assumption that the observed cortical thickness is linearly biased for biological features and nonlinearly biased for dataset (protocol) and brain regions:


$$y_{i\,j\,k}=\alpha_{j\,k}+{\text{X}}_{i\,j}\,\beta_{j\,k}+\varepsilon_{i\,j\,k}.$$

where*y*_*ijk*_ is the observed cortical thickness of *k*-th brain region of *i*-th subject from *j*-th dataset, and **X**_*ij*_ is a 1×*p* vector of biological covariates of *i*-th subject from *j*-th dataset. α_*jk*_ is the average cortical thickness for k-th ROI from *j*-th dataset, and β_*jk*_ is *p*×1 vector of the regression coefficients associated with **X**_*ij*_ for *k*-th ROI. ε_*ijk*_ is the residual term, which cannot be explained by biological covariates, which are assumed to follow the Gaussian distribution εi⁢j⁢k∼N(0,σ)2.

For each dataset and ROI, we calculate an estimator ${\hat{\beta }}_{j\,k}$ of the parameter vector β_*jk*_ using iteratively reweighted least squares (IRLS). The normalized w-score calculation is done by dividing the difference between real cortical thickness value and the predicted value by standard deviation (SD) of the residuals:


$$w_{i\,j\,k}=\frac{y_{i\,j\,k}-{\hat{y}}_{i\,j\,k}}{\text{SD}\,\left({\text{y}}_{j\,k}-{\hat{\text{y}}}_{j\,k}\right)}.$$

#### ComBat

The ComBat harmonization model (Johnson et al., 2007) has similar assumptions as the protocol-specific w-score method. However, instead of fitting MLR model per each dataset, the ComBat harmonization model includes the dataset information to the covariates. Furthermore, it makes the assumption that dataset difference has multiplicative effects as well as additive effects on the data. As a result, the ComBat model describes the observed cortical thickness (*y*) of the *i*-th subject from *j*-th dataset at each region (*k*) as follows:


$$y_{i\,j\,k}=\alpha_{k}+{\text{X}}_{i\,j}\,\beta_{k}+{\text{Z}}_{i\,j}\,{\text{b}}_{k}+\delta_{j\,k}\,\varepsilon_{i\,j\,k},$$

where **X**_*ij*_ is the biological covariates of *i*-th subject from *j*-th dataset, α_*k*_ is the average cortical thickness for k-th ROI, β_*jk*_ is the coefficients associated with **X**_*ij*_ for *k*-th ROI, **Z**_*ij*_ is the dataset indicator of *i*-th subject from *j*-th dataset, **b**_*k*_ is the coefficients associated with **Z**_*ij*_ for *k*-th ROI, and ε_*ik*_ is the residual term, which cannot be explained by biological covariates nor center effects, which is assumed to have mean 0. The parameter δ_*jk*_ describes the multiplicative effect of the *j*-th dataset on *k*-th ROI. For easier understanding of the notations used in Johnson et al. (2007), we rewrote **Z**_*ij*_**b**_*k*_ as γ_*jk*_ as the authors of Fortin et al. (2018) did. For each brain region, we estimated the parameters γ_*jk*_ and δ_*jk*_ using Empirical Bayes, as described in Johnson et al. (2007). The final ComBat harmonized score is defined as follows:


$$y_{i\,j\,k}^{\text{ComBat}}=\frac{y_{i\,j\,k}-{\text{X}}_{i\,j}\,{\hat{\beta }}_{k}-\gamma_{j\,k}^{*}}{\delta_{j\,k}^{*}}+{\text{X}}_{i\,j}\,{\hat{\beta }}_{k}.$$

#### Linear Mixed Effect Model-Based W-Score

The LME model-based w-score models the cortical thickness of a specific subject with the linear bias of biological covariates and the nonlinear bias of the brain region, similar to the protocol specific w-score. However, it is different from the protocol-specific w-score in that the model considers nonlinear bias caused by differences in datasets. LME regards biological features as fixed effects and dataset information as random effects and models the relationship between them and cortical thickness as follows:


$$y_{i\,k}=\alpha_{k}+{\text{X}}_{i}\,\beta_{k}+{\text{Z}}_{i}\,{\text{b}}_{k}+\varepsilon_{i\,k}.$$

In this study, *y*_*ik*_ is the observed cortical thickness of *k*-th brain region of *i*-th subject, **X**_*i*_ is a 1×*p* vector of biological covariates of *i*-th subject, α_*k*_ is the average cortical thickness for k-th ROI, and β_*k*_ is *p*×1 vector of the fixed effects coefficients associated with **X**_*i*_ for *k*-th ROI. **Z**_*i*_ is the dataset indicator of *i*-th subject, which is the biggest point compared with the two methods introduced above. Unlike the *Self-W* method, where the harmonization model had to be created as much as the number of the datasets multiplied by the number of brain regions, the LME model only needs to be trained considering the number of brain regions. **b**_*k*_ is the random effect coefficients associated with **Z**_*i*_ for *k*-th ROI, whic h is assumed to follow the distribution **b**_*k*_∼*N*(0,**D**), and ε_*ik*_ is the residual term, which cannot be explained by biological covariates nor center effects, which is assumed to follow the Gaussian distribution εi⁢k∼N(0,σ)2.

From the above LME model, we may notice the difference between the marginal and conditional mean of *y*_*ik*_. The marginal mean of *y*_*ik*_ is


$$E\,\left(y_{i\,k}\right)={\text{X}}_{i}\,\beta_{k},$$

while the conditional mean of *y*_*ik*_ given **b**_*k*_ is


$$E\,\left(y_{i\,k}\right)={\text{X}}_{i}\,\beta_{k}+{\text{Z}}_{i}\,{\text{b}}_{k}.$$

The fixed-effect parameters β is assumed to be the same for all subjects and must be interpreted in terms of population, while the random effect parameters **b** results in dataset-specific regression, which describes the mean trajectory of a specific dataset.

For each brain region, we calculated the estimator $\hat{\beta }$ of the parameter vector β using the closed-form solution for maximum likelihood (ML):


$$\hat{\beta }={\left(\sum_{i=1}^{n}{\text{X}}_{i}^{Y}\,{\hat{\Sigma }}_{i}^{-1}\,{\text{X}}_{i}\right)}^{-1}\,\sum_{i=1}^{n}{\text{X}}_{i}^{Y}\,{\hat{\Sigma }}_{i}^{-1}\,y_{i},$$

where ${\hat{\Sigma }}_{i}={\text{Z}}_{i}\,\hat{\text{D}}\,{\text{Z}}_{i}^{T}+{\hat{\sigma }}^{2}$. We also calculate the estimator $\hat{\text{D}}$ and $\hat{\sigma }$ of the parameter vector **b** by maximizing the following restricted ML function (ReML) (Verbeke, 1997):


$$l_{R\,e\,M\,L}=\frac{1}{2}\sum_{i=1}^{n}\text{log}|\Sigma_{i}^{-1}|-\frac{1}{2}\sum_{i=1}^{n}{\left(y_{i}-X{}_{i}\hat{\beta }\right)}^{T}\Sigma_{i}^{-1}\left(y_{i}-X{}_{i}\hat{\beta }\right)-\frac{1}{2}\log\sum_{i=1}^{n}X{}_{i}^{T}|\Sigma_{i}^{-1}|X{}_{i},$$

where $\Sigma_{i}={\text{Z}}_{i}\,{\text{DZ}}_{i}^{T}+\sigma^{2}$. There is no closed-form solution for the ReML function; therefore, numerical iterative solvers need to be used. We have implemented the quasi-Newton optimizer provided in MATLAB^2^.

The normalized w-score calculation is carried out by dividing the difference between real cortical thickness value and the predicted value by standard deviation (SD) of the residuals:


$$w_{i\,k}=\frac{y_{i\,k}-{\hat{y}}_{i\,k}}{\text{SD}\,\left({\text{y}}_{k}-{\hat{\text{y}}}_{k}\right)}.$$

**FIGURE 1:**
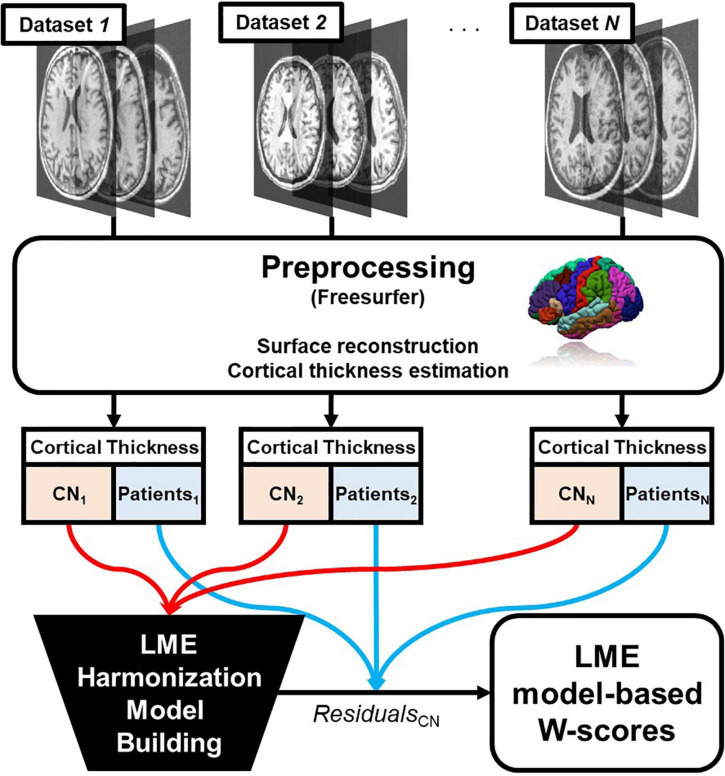
Overall pipeline of the proposed linear mixed effect model harmonization method. Abbreviations: LME, linear mixed effect; CN, cognitive normal.

The process is summarized in Figure 1.

### Center-Effect-Free Harmonization

In our first experiment, we conducted binary classification for dataset prediction over the discovery set before and after the normalization process. The 68-ROI raw cortical thickness values and the corresponding w-score calculated by the LME model were used as input features for classification. The w-scores were corrected for age, sex, years of education, and intercranial volume (ICV). We assumed that biological covariates and center effects would behave like noise in the cortical thickness. However, we believed that there will also be effects that we have not considered, such as atrophy due to diseases. Therefore, we conducted the experiment using only CN subjects to minimize the impact of the disease when testing whether the center effect can be well calibrated.

We used all the CN subjects in each dataset when we trained the harmonization model because the number of CN subjects in some datasets was not enough to be used for training the harmonization model. We thought it would be better to train the model using all the data rather than dividing the dataset into train-test sets in this case. In addition, we hoped that even if we conducted the classification experiment with the harmonization scores calculated based on as much information as possible, we would be able to show whether the classification model proceeds with an ideal performance of nearly 50%.

The classification was carried out using the PCA-LDA framework, which is a well-known classification technique in traditional machine learning field (Zhao et al., 1999; Lu et al., 2003; Cho et al., 2012; Lee et al., 2018; Kim et al., 2019). When there is class imbalance between any two datasets, we undersampled the training data until both the dataset was equal in number. To show that the optimized classifier cannot discriminate harmonized w-scores of each dataset, the model performance was calculated as the test accuracy of the training set. The classification procedure including the undersampling process was randomly repeated 30 times, and we reported the overall mean accuracy.

### Disease-Effect-Preserving Harmonization

As the second experiment, we performed binary classification to discriminate the cognitive normal group and the patient group. Our goal was to show that LME-based w-scores well characterize disease factors even after conversion in cortical thickness. We first compared the classification accuracy of the normal-vs.-disease group for each dataset to show that the w-score did not lose the disease factor at the individual level. We also reported the classification accuracy of another dataset, which we call *merged AD*, where we merged all discovery set except D10, to test if that same results were obtained for the whole normal-vs.-AD group datasets with the center effect corrected. The classification details are equal to that of the section **2.4.**

### Intrasubject Validation

For external validation, we performed an in-subject experiment. We conducted an experiment with a total of 20 images of 10 subjects taken at similar time periods at two different centers. One of these two centers corresponds to D8, and the other is not included in the discovery set. The dataset contains 10 subjects, which is not enough to train the *Self-W* and *ComBat* models, so we cannot calculate the harmonization score for these two methods. However, in the LME model, even datasets that were not used for training can be inferenced to calculate the w-score. Therefore, with the LME model trained on the discovery set, we had to check how well the non-discovery set image features were harmonized when converted to LME model-based w-scores. We compared the differences in mean values of raw cortical thickness and LME model-based w-scores between the two centers. To adjust the variance of the two domains, we divided the cortical thickness by the standard deviation of the normal patient data at D8.

### Scalability of Linear Mixed Effect Model

One of the advantages of using the LME model is that we can make inferences of data with unknown random effect variable. In other words, well-built LME model must be able to calculate w-scores of subjects whose center lack of CN subjects and thus is impossible to build its own w-score calculation model. To show this, we compared the protocol-specific w-scores and LME model-based w-score through the following procedure.

For all the subjects in the discovery set, we calculated the *LME-W* of a single test dataset with all the possible combinations of the other 9 datasets remaining. Then, we obtained the root mean square error (RMSE) between each *LME-W* calculated and the reference *LME-W* of the corresponding dataset. Finally, the RMSE obtained for all combination is averaged according to the number of datasets used when calculating the *LME-W*, as a result we could be able to observe the overall trend of error over the number of datasets used to build an LME model.

## Results

### Center-Effect-Free Harmonization

**FIGURE 2:**
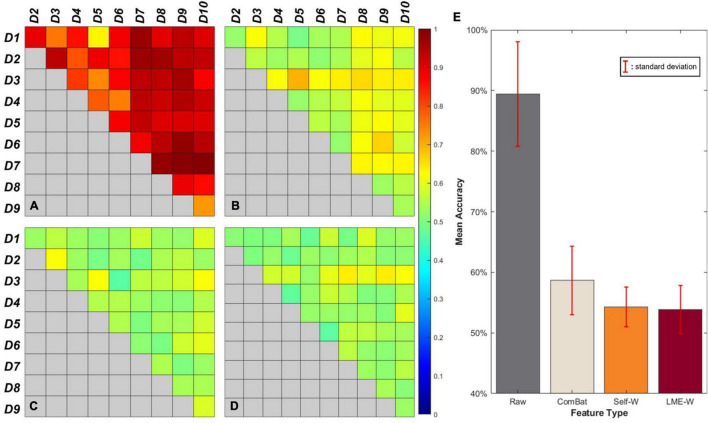
Visual results of binary classification using different harmonization methods. Each cell of the box represents the accuracy of center-wise classification results carried out by **(A)** Raw, **(B)** ComBat, **(C)** Self-W, and **(D)** LME-W. Mean accuracy and standard deviation classification results of each harmonization scores are described as bar graphs at **(E)**.

Figure 2 shows our results of the *center effect-free harmonization* experiment. For the heatmap-looking boxes (Figures 2A–D), the color of each cell represents the mean accuracy of successfully predicting a subject’s data origin. If the experiment is conducted with two datasets from which the center effect is completely removed, the binary classification result of the well-trained classifier will converge to 50%. Therefore, it can be interpreted that the closer the cell of the figure is to green as a whole, the more successfully the center effect is removed.

The bar graph (Figure 2E) shows the average classification accuracy of each score. The classification results of *raw* have an accuracy of 89.5% on average, while the classification results of harmonized scores show dramatically low accuracy, which are 54.3%, 58.6%, and 53.6% on average for *Self-W*, *ComBat*, and *LME-W*, respectively.

### Disease-Effect-Preserving Harmonization

We reported our classification results including accuracy, sensitivity, and specificity in Table 3. Each score showed high average accuracy of 78.7, 83.0, 81.6, and 83.3%, for *raw*, *Self-W*, *ComBat*, and *LME-W*, respectively. In all datasets, the accuracy of classification using *LME-W* is superior to that of *raw* (+ 1.2%p– + 10.3%p). Other harmonization scores also outperform *raw* in accuracy, sensitivity, and specificity. Except for datasets D2, D5, and D6, *LME-W* shows better classification performance than other harmonization scores. For those datasets, *Self-W* was the best for D2 and *ComBat* for D5 and D6. For the “AD” dataset, which merged all datasets including Alzheimer patients, *LME-W* performed better than other scores, with 82.2, 74.1, 82.1, and 76.5% for *LME-W*, *raw*, *Self-W*, and *ComBat*, respectively, in terms of acuity.

**TABLE 3:** Cognitive normal-vs.-patient prediction results before/after normalization.

	Raw			SELF-W			ComBat			LME-W		
				
	Acc.	Sen.	Spe.	Acc.	Sen.	Spe.	Acc.	Sen.	Spe.	Acc.	Sen.	Spe.
D1	0.784	0.874	0.714	0.815	0.904	0.686	0.782	0.859	0.696	**0.832**	**0.911**	**0.722**
D2	0.850	0.879	0.841	**0.917**	**0.929**	**0.895**	0.855	0.921	0.780	0.880	0.895	0.836
D3	0.840	0.882	0.819	0.891	0.941	0.811	0.894	0.941	**0.836**	**0.900**	**0.942**	**0.836**
D4	0.760	0.850	0.690	**0.863**	0.920	0.767	0.862	**0.923**	0.782	**0.863**	0.922	**0.783**
D5	0.836	0.883	0.810	0.849	**0.899**	0.769	**0.859**	0.894	**0.814**	0.848	0.885	0.770
D6	0.807	0.888	0.746	0.839	0.890	0.758	**0.886**	**0.926**	**0.816**	0.847	0.895	0.769
D7	0.621	**0.672**	0.570	0.670	0.669	**0.641**	0.668	0.664	0.586	**0.679**	0.659	0.61
D8	0.827	0.907	0.747	0.850	0.926	0.745	0.793	0.936	0.660	**0.863**	**0.942**	**0.755**
D9	0.830	0.940	0.739	0.874	0.976	0.742	0.867	0.915	**0.770**	**0.879**	**0.987**	0.741
D10	0.716	0.753	0.689	0.769	0.754	0.754	0.736	0.748	0.727	**0.777**	**0.804**	**0.77**
AD	0.741	0.799	0.703	0.821	0.904	0.710	0.765	0.890	0.641	**0.822**	**0.896**	**0.718**

*The bolded values show the highest performance in the corresponding dataset.*

### Intrasubject Validation

**FIGURE 3:**
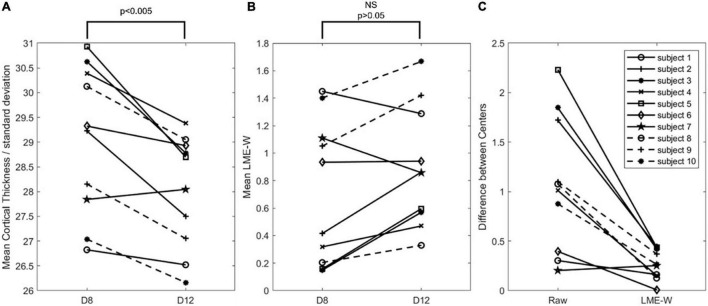
Intrasubject comparison of Raw and LME-W. **(A)** Spaghetti diagram showing mean cortical thickness of each dataset divided by standard deviation of cognitive normal subjects of D8. **(B)** Spaghetti diagram showing mean the LME-based W-score of each dataset. **(C)** Spaghetti diagram showing the difference between two centers of before and after the harmonization. Abbreviations: NS, not significant.

Figure 3 shows that the mean LME model-based w-score of the whole brain is not significantly different between the centers from the same subjects (*p* > 0.05), while the mean cortical thickness of the whole brain is significantly different (*p* < 0.005). To compare the difference in two scales, cortical thickness was divided by the standard deviation of the CN subjects of D8. Compared with raw, the center-to-center difference of LME-W showed an average decrease of 66.1% among the subjects.

### Scalability of Linear Mixed Effect Model

**FIGURE 4:**
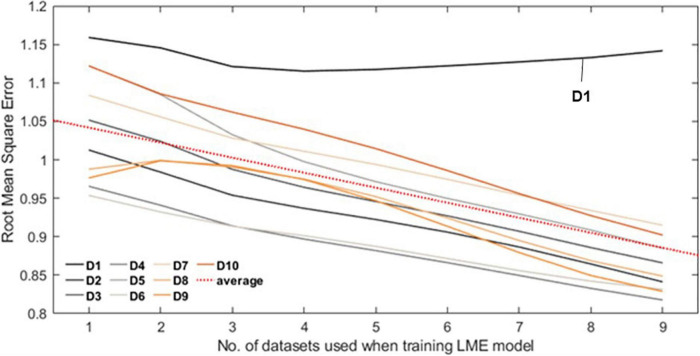
Each line plot represents the root mean square error (RMSE) between the Self-W and LME-W of the datasets. In the experiment, all LME models that were used to calculate the LME-W of a dataset were trained without the corresponding dataset. The red dot line represents the fitted linear plot of average RMSE over all datasets.

Figure 4 shows the RMSE normalized by interquartile range over the number of datasets. Of all datasets, only D1 shows the minimum error when the number of datasets used is 4 and then gradually increases after that. Other datasets show decreasing trend, from 1.043 (no. of dataset used = 1) to 0.888 (no. of datasets used = 9) on average (red dash line).

## Discussion

The LME model framework, which considers samples drawn in a specific population as random effects and analyzes other variables as fixed effects, has been a popular method for dealing with longitudinal data. Using this nature of LME, we were able to find the way to approaching center effects, a problem which is prominent in neuroimaging field. Among the existing studies that applied MLR to solve such incompatibility problem across multiple centers, few studies consider the center of sampling data as a random effect. The LME model showed efficacy in solving this problem.

As the first experiment, we applied the LME model and the existing well-known methods to calculate the harmonization score for 10 datasets to see how well each method corrects the center effect. Binary classification using PCA-LDA framework was performed to check whether machine learning algorithms can distinguish each other’s centers in four cases, namely, raw cortical thickness, protocol-specific w-score, ComBat score, and LME model-based w-score. In this study, the protocol-specific w-scores are obtained based on the CN of each dataset. Since the *Self-W* of each dataset is calculated without being affected by other datasets, the average score of CN in each dataset becomes zero. Therefore, in this study, we assumed that the center effect is sufficiently corrected from *Self-W*, and it may play a role as a gold standard.

The ComBat harmonization score corrects the center effect well compared with the raw cortical thickness, but the LME model-based w-score and protocol-specific w-score show much lower overall binary classification accuracy. Since the protocol-specific w-score was calculated using the CN of each dataset as a reference, in theory, it plays a role of the gold standard in this study. Compared with the protocol-specific w-score, the average accuracy of the LME model-based w-score is slightly close to the baseline accuracy 50%. Even taking into account that the standard deviation of LME model-based w-score is slightly larger than that of the protocol-specific w-score, it can be interpreted that the LME model sufficiently corrects the center effect.

As the second experiment, we conducted binary classification of normal and patient groups to show that the LME model-based w-score is a score that shows the disease effect of individual patients while compensating the center effect. Of all the classification results, the D7 dataset has poor classification accuracy (62.1%–67.9%). The patients with Alzheimer’s disease at the OASIS center (corresponding to the D7 dataset) were 45 patients with clinical dementia rating (CDR) of 0.5 and 12 patients with CDR of 1. Since the AD progression across the dataset has been minor, it would have been difficult to perform the task of classification between CN and patients. However, the remaining datasets showed high classification accuracy regardless of the harmonization procedure, and the average accuracy for each score, excluding the result of D7, corresponds to 80.6, 85.2, 83.7, and 85.4%, for *raw*, *Self-W*, *ComBat*, and *LME-W*, respectively. From this, we found that the LME model-based w-score preserves the disease effect while compensating for the center effect.

An interesting result of the classification between normal and patient groups is that the overall classification accuracies of harmonization scores are higher than that of *raw*. For this result, we believed that the w-score is a more suitable input for this kind of classification task, compared with *raw*, which remains biased to the biological covariates. Also, the results for the *merged AD* dataset are interesting. In the case of *raw*, the accuracy of the *merged AD* dataset (74.1%) was lower than the average accuracy of each dataset, whereas *LME-W* (82.2%) and other harmonization scores (84.1% for *Self-W* and 83.0% for *ComBat*) did not. This shows that merging the data without removing center effect lowers the overall classification and at the same time suggests the possibility that *LME-W* can be used for large-scale experiments by merging the data, which might be more attractive in future big-data analysis.

Furthermore, a notable part of the results of this experiment is that the difference in classification accuracy between *raw* and other harmonic scores varies depending on the dataset. For example, in the case of D3, D4, and Merged AD, the results of *raw* and *LME-W* showed high differences of 6.0%p, 10.3%p, and 8.1%p, respectively, while D5 and D8 showed relatively low differences of only 1.2%p and 3.6%p, respectively. We have not prepared a clear explanation for these differences, and we suspect that the distribution of biological covariates that play an important role in AD pathology, such as Mini-Mental State Exam (MMSE) scores and APOE genetic information, varies different from dataset to dataset.

**FIGURE 5:**
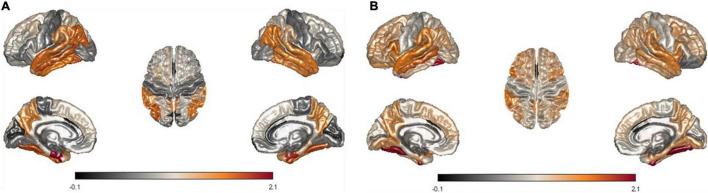
Visualization of mean LME-W over each patient with neurodegenerative disease. Since the w-score represents the atrophy of each region, LME-W identifies the entorhinal cortex, fusiform gyrus, temporal lobe, and inferior parietal lobe as the Alzheimer’s dementia risk area **(A)**. Similarly, fusiform gyrus, precuneus, supramarginal gyrus, and temporal lobe are identified as the Parkinson’s disease risk area **(B)**.

Furthermore, we modeled the average of LME-W for each neurodegenerative disease on the brain surface mesh to conduct qualitative observations of the atrophy pattern according to the disease. The entorhinal cortex (Marzi et al., 2018; Grubman et al., 2019), fusiform gyrus (Chang et al., 2016), temporal lobe (Miller et al., 2013; Wolk et al., 2017), and inferior parietal (Greene et al., 2010), which are areas of frequent atrophy in the brains of Alzheimer’s patients, were identified in Figures 5A,B shows fusiform gyrus (Watanabe et al., 2013; Tard et al., 2015), precuneus (Thibes et al., 2017; Jia et al., 2019), supramarginal (Watanabe et al., 2013), and temporal lobe (Tard et al., 2015), which are frequently atrophy areas in the brain of Parkinson patients. Through the above two cases, it could be seen that the atrophy pattern according to the neurodegenerative disease is well represented by the expression of LME-W.

In addition, through intrasubject experiments, we tried to check whether our harmonization process works well for data, which were not used for the LME model training. When analyzing the MRI of 10 subjects obtained from two different centers, the mean cortical thickness showed a significant difference between centers. However, there was no significant difference when LME model-based w-scores were calculated for the same data. We visualized the difference between the centers before and after harmonization for each individual with spaghetti diagram, and it showed that the difference was reduced except for one subject.

Finally, we used the LME model trained by the whole discovery set as a reference and conducted an experiment to check the scalability of the LME model by measuring the error between the reference and the estimated *LME-W* of the center not used when training the LME model. We have obtained the result that the w-score gets closer to the reference as the number of centers used when learning the LME model increases. As a conclusion together with the previous intrasubject comparison experiment, it can be seen that our LME model can successfully reconcile data not used for training, and that the reconciliation performance improves as the number of datasets used when training the LME model increases.

This fact seems to be very important in future multicohort studies. In many real-world situations, when the number of CN in a certain center is insufficient, the existing methodology will not be able to predict from which distribution the data in the center is sampled. If the proposed LME model is well-trained, this problem can be solved by estimating data distribution through coefficients corresponding to random effects. In addition, from the results obtained above, it could be inferred that if the proposed LME model can calculate the w-score close to the reference for such a center.

There are several limitations to our study, one of which is that we have identified only the presence of harmonization in cortical thickness among the many biomarkers available in neuroimage. In the future, we would like to try harmonization if we obtain datasets for other biomarkers with center effects. Another limitation is that our dataset is heavily skewed to AD among various neurodegenerative descriptions. Although there were also dataset containing patients with PD (D10), the number was too small, and in the future, we would like to conduct experiments with data on various neurodegenerative diseases which comes with cortical atrophies. Finally, the harmonization method using LME can be biased by the imbalance variable between datasets. These biases may be included in random effects removed through LME along with center effects. Therefore, in this study, we cannot say that we have removed only the center effects, and in the future, we will be able to design a more detailed experiment on this problem.

## Data Availability Statement

The datasets used and analyzed during the current study are available from the corresponding author on reasonable request. Requests to access these datasets should be directed to J-KS, jkseong@korea.ac.kr.

## Ethics Statement

The studies involving human participants were reviewed and approved by the Institutional Review Board of Gachon University Gil Medical Center, the Institutional Review Board of Yonsei University Severance Hospital, the Institutional Review Board of the Samsung Medical Center. The participants provided their written informed consent to participate in this study.

## Author Contributions

S-WK, J-KS, and SS: conceptualization. YN, PL, DN, and SS: data curation. SK and S-WK: formal analysis. YN: funding acquisition. SK, S-WK, and J-KS: methodology. SS and J-KS: resources and validation. SS: writing—original draft. J-KS: writing—review and editing. All authors contributed to the article and approved the submitted version.

## Conflict of Interest

The authors declare that the research was conducted in the absence of any commercial or financial relationships that could be construed as a potential conflict of interest.

## Publisher’s Note

All claims expressed in this article are solely those of the authors and do not necessarily represent those of their affiliated organizations, or those of the publisher, the editors and the reviewers. Any product that may be evaluated in this article, or claim that may be made by its manufacturer, is not guaranteed or endorsed by the publisher.

## Footnotes

1
http://surfer.nmr.mgh.harvard.edu

2
mathworks.com
